# Role of C677T and A1298C MTHFR, A2756G MTR and -786 C/T eNOS Gene Polymorphisms in Atrial Fibrillation Susceptibility

**DOI:** 10.1371/journal.pone.0000495

**Published:** 2007-06-06

**Authors:** Betti Giusti, Anna Maria Gori, Rossella Marcucci, Ilaria Sestini, Claudia Saracini, Elena Sticchi, Francesca Gensini, Cinzia Fatini, Rosanna Abbate, Gian Franco Gensini

**Affiliations:** 1 Department of Medical and Surgical Critical Care and Center of Research, Transfer and High Education, “DENOTHE”, University of Florence, Florence, Italy; 2 Department of Clinical Pathophysiology, Section of Medical Genetics, University of Florence, Florence, Italy; 3 Don Carlo Gnocchi ONLUS Foundation, Centro S. Maria agli Ulivi - IRCCS, Florence, Italy; DER Neurogenetics, National Institute of Neurological Disorders and Stroke, United States of America

## Abstract

**Background:**

Hyperhomocysteinemia has been suggested to play a role in the NonValvular Atrial Fibrillation (NVAF) pathogenesis. Polymorphisms in genes coding for homocysteine (Hcy) metabolism enzymes may be associated with hyperhomocysteinemia and NVAF.

**Methodologies:**

456 NVAF patients and 912 matched controls were genotyped by an electronic microchip technology for C677T and A1298C MTHFR, A2756G MTR, and -786C/T eNOS gene polymorphisms. Hcy was determined by an immunoassay method.

**Principal Findings:**

The genotype distribution of the four polymorphisms as well as genotype combinations did not differ in patients and controls. Hcy was higher in patients than in controls (15.2, 95%CI 14.7–15.7 vs 11.3, 95%CI 11.0–11.6 µmol/L; p<0.0001). In both populations, a genotype-phenotype association (p<0.0001) between Hcy and C677T MTHFR polymorphism was observed; in controls a significant (p = 0.029) association between tHcy and −786C/T eNOS polymorphism was also observed. At the multivariate analysis the NVAF risk significantly increased in the upper quartiles of Hcy compared to the lowest: OR from 2.8 (1.68–4.54 95%CI) in Q2 to 12.9 (7.96–21.06 95%CI) in Q4.

**Conclusions:**

Our data demonstrated the four polymorphisms, although able, at least in part, to affect Hcy, were not associated with an increased risk of NVAF *per se* or in combination.

## Introduction

Nonvalvular atrial fibrillation (NVAF) is the most common arrhythmia in clinical practice [1]. The molecular basis for the development of structural remodeling of fibrillating human atria is still a matter of debate and investigation. *“Ex vivo”* studies have shown that NVAF is associated with enhanced activity of matrix metalloproteinases (MMPs) [2] and tissue inhibitor of metalloproteinases (TIMPs) [3] at the human atrium level and in particular with an increase in the expression of a disintegrin and metalloproteinases (ADAM) 10 and ADAM15 [4]. In animal models and in vitro human experiments it has been shown that MMPs are activated by Hcy in both endothelial cells and arterial media [5]–[8]. We demonstrated a significant association of elevated total homocysteine (tHcy) levels with the presence of NVAF [9]. This datum strongly suggested the possible role of hyperhomocysteinemia (HHcy) as new factor involved in the pathogenesis of NVAF. Homocysteine is a highly reactive, sulfur-containing amino acid formed as a by-product of the essential amino acid methionine. It is estimated that 5 to 7% of the general population have mild to moderate HHcy. Mild HHcy may result from both acquired (e.g. vitamin deficiencies) and genetic influences [10], [11]. Several genetic polymorphisms in gene coding for enzymes involved in the Hcy metabolism are demonstrated or suspected to be associated with HHcy [C677T and A1298C in 5,10-methylenetetrahydrofolate reductase (MTHFR), A2756G in methionine synthase (MTR)]. For C677T and A1298C MTHFR polymorphisms, the variant allele is associated with reduced enzyme activity in vitro [12], [13]. For the A2756G MTR polymorphism, functional data are limited and inconsistent [14]. Moreover, we previously demonstrated that −786 T/C endothelial nitric oxide synthase (eNOS) polymorphism, which is associated with a reduction in the eNOS gene promoter activity, affects plasma Hcy concentrations mildly but significantly and independently [15].

Aim of this study was to investigate whether C677T and A1298C MTHFR, A2756G MTR and −786 C/T eNOS gene polymorphisms *per se* or in combination are associated with NVAF. Our data demonstrated that the 4 polymorphisms, although able, at least in part, to alter tHcy levels, were not associated, *per se* or in combination, with an increased risk of NVAF.

## Materials and Methods

### Subjects

We studied 456 consecutive chronic NVAF patients (271 men, 185 women) on oral anticoagulation, recruited from September 2002 to September 2005. All patients underwent an electrocardiogram, transthoracic echocardiography, and carotid artery ultrasound study. Exclusion criteria for patients were the presence of hyperthyroidism or renal failure (serum creatinine>2.0 mg/dL).

The control population comprised 912 matched (for age and sex) healthy subjects (males 542, females 370) (blood donors and partners or friends of the patients) from the same geographical area. Exclusion criterion for the controls was a history of cardiovascular disease or venous thromboembolic events evaluated by a structured questionnaire to identify symptom-free controls and to exclude subjects who were suspected of having any form of vascular disease. No subject had abnormal liver or renal function.

Patients and controls gave their informed consent and the study was approved by the Ethical Committee of the Careggi University Hospital (Florence, Italy). Patients and controls were entirely unrelated.

The presence of traditional cardiovascular risk factors and risk factors for ischemic complications was assessed on the basis of patients' interview, echocardiography data and hospital records. Dyslipidemia was defined according to the Third Report of the National Cholesterol Education Program; hypertension in the presence of blood pressure values above 130/80 mmHg and/or an antihypertensive treatment and diabetes in agreement with the criteria of the American Diabetes Association.

### Blood collection and Hcy measurement

Venous blood was collected after an overnight fasting between 8 am and 10 am. For Hcy measurement whole venous blood was collected in tubes containing ethylenediaminotetracetate (EDTA) 0.17 mol/L, immediately put in ice and centrifuged within 30 minutes at 4°C (15000×g for 15 min). The supernatant was stored in aliquots at −80°C until assay. The plasma levels of total Hcy were determined by an immunoassay method (FPIA assay, IMX system, Abbott).

### DNA Extraction

Genomic DNA was isolated from peripheral blood leukocytes by using the Flexi Gene DNA kit (QIAGEN GmbH, Germany).

### C677T MTHFR, A1298C MTHFR and A2756G MTR polymorphisms detection by electronic microchip

We analyzed 3 DNA polymorphisms (C677T MTHFR, A1298C MTHFR and A2756G MTR) by using the NanoChip Molecular Biology Workstation and the NanoChip cartridge, a 10×10 array (100 pads). Briefly, in the following paragraph we reported the protocols extensively described in a previous paper [16].

#### Probe design

MTHFR and MTR gene sequences were obtained from GeneBank (www.ncbi.nlm.nih.gov, accession number AY338232 and AL359259, respectively). For each polymorphism we designed a set of probes consisting of a forward and a reverse oligonucleotide for the polymerase chain reaction (PCR) amplification, two reporter oligonucleotides (one, labeled with Cy3, specific for the wild-type nucleotide, the other, labeled with Cy5, specific for the mutant nucleotide), and one stabilizer oligonucleotide. All the designed oligonucleotides are reported in Table 1.

**Table 1:** PCR and hybridization oligonucleotide designs for electronic microchip analysis

SNP	Type of oligonucleotides	Sequences	Tm (°C)	Ta (°C)	Tb (°C)
MTHFR C677T	Forward PCR	5′biotinTGAAGGAGAAGGTGTCTGCGGGA3′	63.6	64	
	Reverse PCR	5′AGGACGGTGCGGTGAGAGTG3′	62.5		
	Stabilizer	5′CTCCCGCAGACACCTTCTCCTTCA3′	63.9		36
	Reporter WT	5′Cy3TGATGAAATCGG3′	33.3		
	Reporter MUT	5′Cy5ATGATGAAATCGA3′	33.7		
MTHFR A1298C	Forward PCR	5′biotinCAAGGAGGAGCTGCTGAAGA3′	57.7	64	
	Reverse PCR	5′CCACTCCAGCATCACTCACT3′	57.7		
	Stabilizer	5′CTTCACTGGTCAGCTCCTCCCCCCAC3′	67.8		41
	Reporter WT	5′Cy3-TCAAAGACACTTT3′	34.1		
	Reporter MUT	5′Cy5TCAAAGACACTTG3′	35.5		
MTR A2756G	Forward PCR	5′CATGGAAGAATATGAAGATATTAGAC3′	50.9	57	
	Reverse PCR	5′biotinGAACTAGAAGACAGAAATTCTCTA3′	51.1		
	Stabilizer	5′CCATTATGAGTCTCTCAAGGTAAGTGGTAGAAAC3′	61.6		38
	Reporter WT	5′Cy3GATATTAGACAGGA3′	33.9		
	Reporter MUT	5′Cy5ATATTAGACAGGG3′	32.1		

Tm = melting temperature; Ta = annealing temperature; Tb = optimal temperature for thermal stringency; SNP = single nucleotide polymorphism.

#### Sample amplification

To genotype the 3 polymorphisms, 3 DNA sequences were amplified by PCR. In Table 1 the annealing temperature for each DNA fragment is reported.

#### Sample preparation

The 3 amplicons of each subject were pooled and desalted using the Nucleo Fast 96 well plates (Macherey-Nagel). Samples were eluted in 55µl of deionized water. Thirty µl of amplicon pools were mixed with 30 µl of 100 mmol/L histidine and transferred to a 96-well plate.

#### Addressing of amplicons on electronic chip

The Loader was programmed to electronically address each amplicon pool to specific pads on the cartridge.

#### Hybridization, stripping procedures and data analysis

One set of stabilizer and reporter oligonucleotides at a time was hybridized to the NanoChip cartridge. Reporter oligonucleotides were mixed at 0.5 µmol/L each in high-salt buffer (50 mmol/L sodium phosphate pH 7.4, and 500 mmol/L NaCl) in combination with 1 µmol/L of stabilizer oligonucleotide. After rinsing, the cartridge was placed in the NanoChip Reader where a specific thermal stringency was applied. The specific temperatures used for each polymorphism are reported in Table 1. After each hybridization and scanning procedure, a stripping procedure was performed to remove reporters, and to allow the hybridization with another set of probes. The reporters were stripped by rising temperature 15°C above the Tm of reporters. In order to study all 3 polymorphisms, we performed 3 hybridization/stripping steps. The software of the System directly assigned the genotype to each sample.

### eNOS −786T>C polymorphism detection

The *eNOS* −786T>C polymorphism was analyzed by PCR with restriction fragment length polymorphism analysis. PCR was performed with the primers 5′-GTGTACCCCACCTGCATTCT-3′ and 5′-CCCAGCAAGGATGTAGTGAC-3′, and DNA (100 ng) was amplified at an annealing temperature of 60°C. The PCR product (306 bp) was digested with *Nae*I restriction enzyme (Turbo Nae I; Promega) to obtain fragments of 225 and 81 bp.

### Statistical analysis

Statistical analysis was performed using the 11.5 SPSS software. Differences in characteristics between NVAF patients and controls were determined by using the t-test or Chi-square test. Log-transformed values for Hcy were used in the analyses, and back transformed for data presentation. Unless otherwise indicated, data are given as geometric means and 95% Confidence Interval (CI). Adjusted Hcy mean values for C677T MTHFR, A1298C MTHFR, A2756G MTR and −786 C/T eNOS genotypes were estimated from linear models adjusted for sex, age and creatinine. For the risk of NVAF associated with Hcy levels, we classified the data into quartiles based on the distribution of this parameter among patients and controls (Q1:≤9.7; Q2:9.8–12.1; Q3:12.2–16.0; Q4:≥16.1 µmol/L). Univariate logistic regression analysis was used to describe the relation of Hcy and MTHFR, MTR and eNOS polymorphisms with NVAF. To perform the multivariate analyses, logistic regression analysis was used with NVAF as the dependent variables and age, sex, traditional cardiovascular risk factors, MTHFR, MTR and eNOS polymorphisms and Hcy as the independent variables. Odds ratios (OR) were adjusted for the potential confounding variables which were associated with NVAF with a p value<0.20 in the univariate analysis. Variables that resulted not to be associated with the outcome were removed from the final most parsimonious regression model through a backward selection algorithm. All OR are given with their 95% CI. A value of p<0.05 was chosen as the cut-off level for statistical significance. The Bonferroni correction was used for multiple testing by multiplying the nominal *p*-value of each test by the number of tests conducted.

## Results

The clinical and laboratory characteristics of patients and control subjects are reported in Table 2.

**Table 2:** Clinical and laboratory characteristics of patients and control subjects

Characteristic	Patients (n = 456)	Controls (n = 912)	
Age (y)	75 (21–98)	75 (21–98)	ns
Sex (M/F)	271/185	542/370	ns
Hypertension (%)	265 (58.1)	379 (41.6)	<0.0001
Diabetes mellitus (%)	50 (13.6)	37 (4.1)	<0.005
Dyslipidemia (%)	139 (30.5)	243 (26.6)	0.077
Smoking habit (%)	155 (34.0)	185 (20.3)	<0.0001
History of CAD (%)	58 (13.6)	–	
LVEF<50% (%)	156 (36.6)	–	

Age is expressed as median (range). Other data are expressed as n (%). CAD, coronary artery disease; LVEF, left ventricular ejection fraction. History of CAD was an exclusion criterium for control subjects. ns = not statistically significant.

Among the traditional risk factors, the prevalence of hypertension, smoking habit and diabetes was significantly higher in patients than in controls (Table 2).

Total Hcy plasma levels were significantly higher in NVAF patients than in controls (geometric mean 15.2, 95% CI 14.7–15.7 vs 11.3, 95% CI 11.0–11.6 µmol/L; p<0.0001 adjusted for sex, age and creatinine).

Table 3 shows C677T MTHFR, A1298C MTHFR, A2756G MTR and −786 C/T eNOS polymorphism genotype distributions and tHcy levels according to genotypes in patients and controls.

**Table 3:** Genotype distributions and tHcy plasma levels according to genotype in patients and controls

Gene Polymorphism	Controls N (%)	tHcy µmol/L	Patients N (%)	tHcy µmol/L
MTHFR C677T				
CC	225 (24.7)	10.7 (10.2–11.2)	133 (29.2)	13.1 (12.4–13.8)
CT	479 (52.5)	10.7 (10.4–11.1)	222 (48.7)	15.4 (14.9–16.7)
TT	208 (22.8)	13.6 (12.9–14.3)	101 (22.1)	17.9 (16.8–19.1)
p for trend		<0.0001		<0.0001
MTHFR A1298C				
AA	423 (46.4)	11.9 (11.5–12.4)	224 (49.1)	15.9 (15.2–16.6)
AC	407 (44.6)	10.8 (10.4–11.2)	196 (43.0)	14.8 (14.1–15.5)
CC	82 (9.0)	11.0 (10.1–12.0)	36 (7.9)	13.2 (11.8–14.8)
p for trend		ns		ns
MTR A2756G				
AA	628 (68.9)	11.4 (11.1–11.8)	319 (70.0)	15.1 (14.64–15.7)
AG	265 (29.1)	11.1 (10.6–11.6)	128 (28.0)	15.2 (14.3–16.7)
GG	19 (2.1)	11.7 (9.8–14.1)	9 (2.0)	17.4 (13.9–21.8)
p for trend		ns		ns
−786 C/T eNOS				
TT	283 (31,0)	11.3 (10.7–12.1)	132 (29,0)	15.9 (14.7–17.1)
TC	432 (47,4)	11.6 (10.7–12.5)	239 (52,3)	15.6 (14.8–16.5)
CC	197 (21.6)	12.8 (12.1–13.5)	85 (18,7)	15.1 (14.2–16.5)
p for trend		0.029		ns

ns = not statistically significant.

The C677T MTHFR, A1298C MTHFR, A2756G MTR and −786 C/T eNOS genotype distributions were in Hardy-Weinberg equilibrium in patients and controls.

The genotype distributions of the 4 polymorphisms were not different in NVAF patients in comparison to controls.

**Figure 1:**
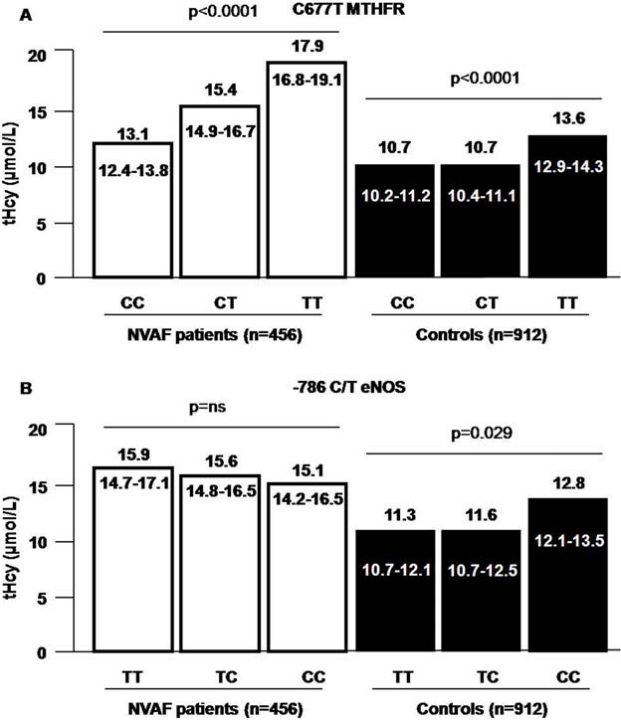
tHcy plasma levels according to C677T MTHFR genotypes (Figure 1A) and −786 C/T eNOS genotypes (Figure 1B) in patients and controls.

In patients and controls a significant genotype-phenotype association (p<0.0001) between tHcy levels and C677T MTHFR polymorphism was observed (Table 3 and Figure 1). A significant genotype-phenotype association between tHcy levels and −786 C/T eNOS polymorphism in controls (p = 0.029), but not in patients, was also observed (Table 3 and Figure 1). No significant genotype-phenotype association between tHcy levels and A1298C MTHFR or A2756G MTR polymorphisms in controls were observed (Table 3), whereas only a trend was found in patients (Table 3). In particular, in patient group, subjects with 1298AA and 1298AC MTHFR and 2756GG MTR genotypes showed higher levels of tHcy (Table 3).

Both in patient and control subjects, the combined genotypes 677TT/1298AC, 677TT/1298CC and 677CT/1298CC were not observed, indicating the linkage disequilibrium between these two polymorphisms in our populations. The distributions of the possible combinations of the two common polymorphisms of the MTHFR gene were not different in NVAF patients in comparison to controls (Table 4). As concerns tHcy, it was significantly higher in 677TT/1298AA combination with respect to all the other possible combinations both in patients and controls (p<0.001 and p<0.0001, respectively). Moreover, in patient group, a statistical difference in tHcy levels among 677CT/1298AC and 677CC/1298AC (p = 0.044) and 677CC/1298AA (p = 0.005), and between 677CT/1298AA and 677CC/1298AA (p = 0.024) was observed.

**Table 4:** Genotype combination distributions and tHcy plasma levels according to genotype combination in patients and controls

MTHFR C677T	MTHFR A1298C	Controls N (%)	tHcy µmol/L	Patients N (%)	tHcy µmol/L
TT	AA	208 (22.8)	13.5 (12.9–14.3)	102 (22.3)	17.9 (16.8–19.1)
CT	AC	302 (33.1)	10.8 (10.3–11.2)	132 (28.9)	15.5 (14.7–16.4)
CT	AA	177 (19.4)	10.7 (10.1–11.4)	90 (19.7)	15.2 (14.2–16.3)
CC	CC	82 (9.0)	11.0 (10.1–11.9)	36 (7.9)	13.2 (11.9–14.7)
CC	AC	105 (11.5)	10.8 (10.0–11.6)	63 (13.8)	13.4 (12.3–14.5)
CC	AA	38 (4.2)	9.9 (8.7–11.1)	33 (7.2)	12.3 (11.0–13.8)

The analysis of the distribution of all the possible combination among C677T and A1298C MTHFR, A2756G MTR and −786 C/T eNOS genotypes between patients and controls did not showed significant differences. The analysis of the effect of the combinations of all the 4 studied polymorphisms demonstrated that the A2756G MTR and −786 C/T eNOS polymorphisms also did not interact with the C677T MTHFR polymorphism, in patients and controls, in influencing tHcy levels (data not shown).

At the multiple analysis (adjusted for sex, age, creatinine levels, hypertension, diabetes, dyslipidemia, smoking habitus, history of CAD, polymorphisms), the risk of NVAF significantly increased in the Q2, Q3 and Q4 of Hcy with respect to Q1: with an OR increasing from 2.8 (1.68–4.54 95% CI) in Q2 to 12.9 (7.96–21.06 95% CI) in Q4 (Table 5). In spite of the influence of the C677T MTHFR polymorphism on Hcy levels, this polymorphism was not associated with the risk of NVAF (Table 5).

**Table 5:** Odds ratios for NVAF according to Hcy quartiles, C677T MTHFR and −786 C/T eNOS polymorphism and other traditional factors known influenced Hcy plasma levels

	Multivariate analysis OR (95% CI)	p*
Hcy		
Q1	1	
Q2	2.8 (1.68–4.54)	<0.0001
Q3	6.6 (4.09–10.67)	<0.0001
Q4	12.9 (7.96–21.06)	<0.0001
C677T MTHFR	0.8 (0.56–1.12)	0.184
−786 C/T eNOS	0.9 (0.68–1.43)	0.945
Hypertension	1.9 (1.46–2.57)	<0.0001
Dyslipidemia	1.4 (1.00–1.86)	0.048
Smoking habit	2.4 (1.68–3.32)	<0.0001
Diabetes mellitus	3.23 (2.56–7.56)	<0.0001

* Adjusted for age, sex, traditional cardiovascular risk factors, polymorphisms and homocysteine (Hcy) quartiles (Q).

## Discussion

In the present study we have evaluated the role of 4 polymorphisms known or suspected to influence Hcy plasma levels in determining susceptibility to NVAF. At this purpose we investigated a large population of consecutive NVAF patients. Our data demonstrated that the 4 polymorphisms (C677T and A1298C MTHFR, A2756G MTR and −786 C/T eNOS polymorphisms), although able, at least in part, to alter tHcy levels, were not associated, *per se* or in combination, with an increased risk of NVAF.

Recently our group demonstrated a significant association between elevated Hcy levels and the presence of NVAF, suggesting a role for HHcy as new factor involved in the pathogenesis of atrial fibrillation [9]. In that study we did not find an association between C677T MTHFR polymorphism *per se* and NVAF [9], but those results were not conclusive, because the number of patients examined was not sufficient to definitively exclude this hypothesis. Moreover, we did not test the possibility that the C677T MTHFR polymorphism could represent a risk factor for NVAF by interacting with other polymorphisms involved in the Hcy metabolism.

Therefore, we studied 4 polymorphisms, three in genes directly involved in Hcy metabolism (C677T and A1298C MTHFR and A2756G MTR) and one in another gene indirectly involved as determinant of Hcy levels (−786 C/T eNOS).

In vitro data showed that C677T and A1298C MTHFR polymorphisms are associated with a reduced enzyme activity [12], [13]. Moreover, several *in vivo* studies demonstrated an association between C677T MTHFR polymorphism and increased tHcy plasma levels in healthy subjects and patients affected by a number of cardiovascular diseases, especially in subjects with low folate levels [17]–[19].

Limited and inconsistent data on the role of A1298C MTHFR and in particular A2756G MTR polymorphism as determinant of tHcy plasma levels are available. Recent data from our group demonstrated on a large population of young healthy subjects that −786 T/C eNOS polymorphism is a mild but independent determinant of tHcy levels [15].

In the present study, as expected, a significant genotype-phenotype association between tHcy levels and C677T MTHFR polymorphism in NVAF patients and controls was observed. A significant genotype-phenotype association between tHcy levels and −786 C/T eNOS polymorphism in these older control subjects was also confirmed, but not observed in NVAF patients. It has to be underlined that it is the second report showing the genotype-phenotype association between −786 C/T eNOS polymorphism and tHcy levels in a large healthy population [previous work [15]:1287 healthy subjects, median age = 60 years, range = 20–78 years; present work: 972 healthy subjects, median age = 75 years, range = 21–98 years].

As concerns the other two polymorphisms, although higher tHcy levels could be observed in NVAF patients carrying the 1298AA MTHFR and the 2756GG MTR genotypes, a significant genotype-phenotype association was not demonstrated in both patients and controls.

In spite of these observations, the evaluation of the possible role of genotype combinations in determining tHcy levels did not identify any interactions among the 4 studied polymorphisms.

As concerns the two MTHFR polymorphisms investigated, the distribution of the combined genotypes suggests that the A1298C MTHFR polymorphism is genetically linked with the C677T MTHFR polymorphism. Previous studies [13], [20]–[22] indicated that the 677T and 1298C alleles do not or very rarely exist in cis (on the same chromosome), suggesting that these two polymorphisms arose independently in separate chromosomes and no recombination had occurred due to short physical distance (2.1 kb) between them. An alternative explanation is that there was a selection against cis because of a severely adverse phenotype. The latest hypothesis was not confirmed through an in vitro study by site-directed mutagenesis, which demonstrated cis did not confer a severe phenotype [23].

These data also explain at least in part the observed higher tHcy levels in 1298AA MTHFR subjects with respect to the other 1298AC and CC genotypes. In fact, due to the linkage disequilibrium, all the 677TT MTHFR subjects belong to the group of subjects with the 1298AA genotype.

Data of the present study, conducted in a different and larger NVAF population with respect to the previous published work on NVAF [9], confirmed the observed strong association between elevated tHcy levels and the presence of NVAF. Interestingly, at the multivariate analysis, the risk of NVAF significantly increased in the three upper quartiles of Hcy with respect to the lowest quartile: with an OR increasing from 2.8 to 12.9. This datum suggests a gradient in which at the increase of Hcy levels corresponds a proportional increase in the estimated risk of NVAF.

Even if associations do not prove causality, this evidence together with that of the previous work [9] (on a total number of 766 patients: 310 previous work and 456 present work) indicates a possible independent role of elevated Hcy levels as a risk factor for NVAF, in a model adjusted for age, sex, and the traditional risk factors for NVAF.

Atrial fibrillation usually occurs in the context of an atrial substrate produced by alterations in atrial tissue properties referred to as remodeling. Increased levels of Hcy might be implicated in the remodeling of the extracellular matrix of the cardiac wall by direct or indirect action. Studies in animal models demonstrated that HHcy could induce marked remodeling of the extracellular matrix by inducing elastolysis through the activation of MMPs [5], [7], [8]. In addition, Hcy may directly affect extracellular matrix components by interfering with intra- and/or inter-molecular disulfide bonds through disulfide exchange, or binding to free sulphydril groups [24], [25].

We should consider the hypothesis that high Hcy levels are either a consequence of NVAF or just a marker of other diseases and/or deficiencies of B vitamins, which can, by themselves, be responsible for abnormalities that increase the risk of NVAF independently of Hcy levels.

Importantly, the association between Hcy and NVAF does not stem from the presence of coronary artery disease as it persists at the multivariate analysis adjusted also for history of coronary artery disease. As far as vitamin status is concerned, a limitation of this study is the lack of information about this issue.

Due to the high number of genes that could modulate plasma Hcy levels [26], we cannot role out the need to evaluate the interaction among a higher number of polymorphisms in the same and different genes. Together with the observation that increased tHcy levels associated with the increased risk of NVAF could be determined by non-genetic factors, we should take into account that the advantage due to polymorphisms in term of protection against cancers [14], [27], [28] in this elder population could, at least in part, mask the contribution of these polymorphisms to the risk for NVAF that is so strong for the increased tHcy levels.
